# Wearable super-resolution muscle–machine interfacing

**DOI:** 10.3389/fnins.2022.1020546

**Published:** 2022-11-17

**Authors:** Huxi Wang, Siming Zuo, María Cerezo-Sánchez, Negin Ghahremani Arekhloo, Kianoush Nazarpour, Hadi Heidari

**Affiliations:** ^1^Microelectronics Lab, James Watt School of Engineering, The University of Glasgow, Glasgow, United Kingdom; ^2^Neuranics Ltd., Glasgow, United Kingdom; ^3^School of Informatics, The University of Edinburgh, Edinburgh, United Kingdom

**Keywords:** electrical impedance tomography, electromyography, forcemyography, human-computer interface, magnetomyography, muscle-machine interface, super-resolution, wearable sensors

## Abstract

Muscles are the actuators of all human actions, from daily work and life to communication and expression of emotions. Myography records the signals from muscle activities as an interface between machine hardware and human wetware, granting direct and natural control of our electronic peripherals. Regardless of the significant progression as of late, the conventional myographic sensors are still incapable of achieving the desired high-resolution and non-invasive recording. This paper presents a critical review of state-of-the-art wearable sensing technologies that measure deeper muscle activity with high spatial resolution, so-called super-resolution. This paper classifies these myographic sensors according to the different signal types (i.e., biomechanical, biochemical, and bioelectrical) they record during measuring muscle activity. By describing the characteristics and current developments with advantages and limitations of each myographic sensor, their capabilities are investigated as a super-resolution myography technique, including: (i) non-invasive and high-density designs of the sensing units and their vulnerability to interferences, (ii) limit-of-detection to register the activity of deep muscles. Finally, this paper concludes with new opportunities in this fast-growing super-resolution myography field and proposes promising future research directions. These advances will enable next-generation muscle-machine interfaces to meet the practical design needs in real-life for healthcare technologies, assistive/rehabilitation robotics, and human augmentation with extended reality.

## Introduction

**FIGURE 1:**
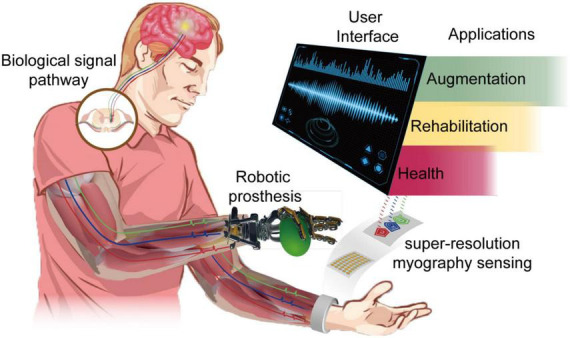
Schematic illustration of wearable super-resolution myography and its applications.

Myography measures muscle activity, which has become essential to modern healthcare, assistive/rehabilitation, and human augmentation technologies (Chowdhury et al., 2013; Mukhopadhyay, 2015; Cheok et al., 2019; Simao et al., 2019; Xiao and Menon, 2019; Mahmud et al., 2020; Zong et al., 2020; Nsugbe, 2021b). Conventional myography techniques include recording the force, known as forcemyography (FMG), or electrical potential, known as electromyography (EMG). These myographic sensors are primarily laboratory-based and entail placing an extensive electrode/wire set up on the skin for the duration of a measurement. In recent years, the development of wearable devices has opened a realm of opportunities for recording muscle signals on a long-term basis without limiting individual physical activities. Additionally, modern CMOS technologies enable the compact and micro-scale design and fabrication of sensors in a mass and low-cost way with many sensing units integrated into a small area, allowing higher signal resolution. Such developments facilitate various muscle–machine interface (MMI) applications, including health monitoring of neuromuscular disorders, control for assistive/rehabilitation robotics, and human augmentation for extended/virtual reality, as conceptualized in Figure 1 (Barry et al., 1990; Xiao and Menon, 2014; Sadikoglu et al., 2017; Sushkova et al., 2018; Grushko et al., 2020).

The market for wearable myography-based MMI applications is experiencing dramatic growth. There is an estimated market of $7.24 billion by 2026 for MMI devices concerning different applications such as extended reality, healthcare, and education (Mordor Intelligence, 2022). Apple Watch’s newest feature, Assistive Touch, can detect muscle activity through an optical device that allows users to perform basic select, swipe, and confirm operations without touching the screen. Alphabet Inc. (previously Google) and Meta Platforms, Inc. (previously Facebook) have invested in muscle-based technologies and acquired well-known myoelectric startups North. Inc (formerly Thalmic Labs) and CTRL-Labs, respectively.

Muscle machine interface technology has been a trending topic in the last 5 years, with more than 3,000 papers published yearly, by searching keywords on the Web of Science. There are several excellent review articles in the literature that have studied and discussed the hardware implementation and algorithms for different MMI-based applications in hand gesture recognition (Cheok et al., 2019; Simao et al., 2019; Grushko et al., 2020), prosthetic control (Grushko et al., 2020; Nsugbe, 2021b), facial movement recognition (Chowdhury et al., 2013; Khoshmanesh et al., 2021), biomedical image (Zong et al., 2020; Lin et al., 2022), and healthcare (Chowdhury et al., 2013; Wang Y. et al., 2019; Khoshmanesh et al., 2021). Summarized from the literature, the trend behind MMI development is to have better signal qualities and features for applications, which requires allocating signals to muscles with higher accuracy. The high channel density of a sensor system has been proven to improve the overall accuracy in applications due to higher spatial resolution and the ability to differentiate proximal features (Radmand et al., 2016; Grushko et al., 2020; Lei et al., 2021). However, the spatial resolution does not remain consistent in terms of depth because MMI sensing technologies have different capabilities to detect deep muscles (Grushko et al., 2020; Nsugbe, 2021b). The potential of wearable MMI for sensing deep muscle with high resolution, considered super-resolution, was never reported in previous reviews.

Compared to the reviews mentioned above, which focus more on specific applications of MMI sensors, this review discusses conventional and novel myography sensing methods from the view of muscle signal form. Considering the signal forms different MMI sensors detect, it is possible to estimate whether this technology is suitable for high-resolution measurement with a deep detection range, referred to as super-resolution in this paper. This paper summarized the state-of-the-art of these myography techniques in each modality and explored their possibility for a super-resolution. The aim here is to provide a better understanding of the practical significance and implications of these myography methods and why some modalities can achieve super-resolution. The rest of this paper is structured into six sections as follows. Section “Muscle–machine interfaces” presents various signal forms during muscle contraction, and how they are measured. Sections “Biomechanical sensing interfaces,” “Biochemical sensing interfaces, “and “Bioelectrical sensing interfaces” introduce different myography sensing technologies, respectively. Section “Discussion and outlook” compares these sensing technologies in terms of vulnerability and detectability as the super-resolution myography, and the future directions and possible research topics are discussed. Section “Conclusion” concludes the advantages and disadvantages of different myographic sensors for super-resolution MMI.

## Muscle–machine interfaces

Muscles are natural amplifiers of the neural drive (Ruff et al., 2010). Thus, with advanced signal analysis methods, e.g., motor unit decomposition and machine learning, muscle signals can be used as a control source in various MMIs, e.g., prostheses (Naeem et al., 2012; Bergmeister et al., 2017), wheelchairs (Jang et al., 2016), exoskeleton (Singh et al., 2012; Kawase et al., 2017; Lyu et al., 2019), and human–robot collaboration (Melcer et al., 2018), Compared with the brain–machine interfaces (Grush, 2016), an MMI can obtain cleaner motor and intention-related signals in terms of signal-to-noise ratio (SNR) (Grush, 2016).

**FIGURE 2:**
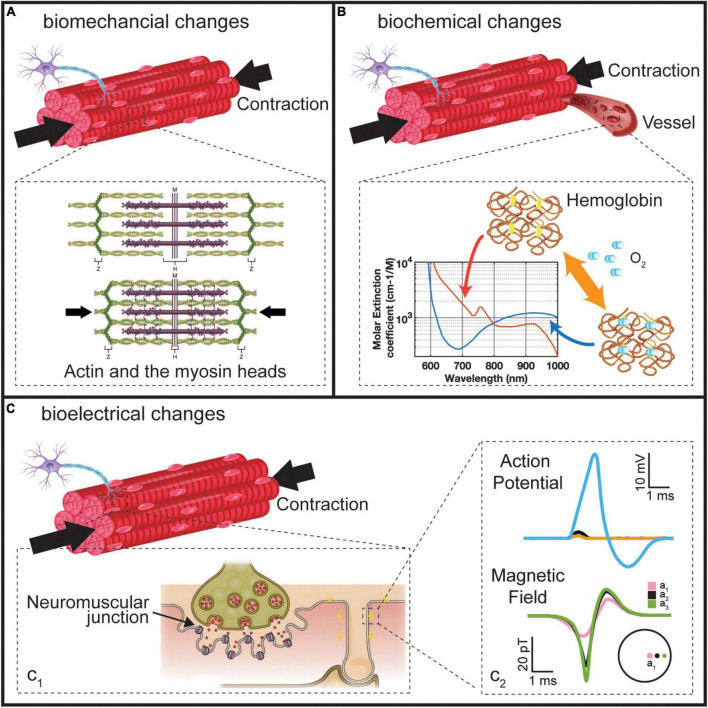
Three changes during the muscle contraction. **(A)** The biomechanical changes. Reproduced under terms of the CC-BY license (OpenStax, 2022). Copyright 2022, OpenStax College, published by Rice University. **(B)** Biochemical changes. **(C)** Bioelectrical changes. Reproduced under terms of the CC-BY license (Ch. 10 Introduction – Anatomy and Physiology | OpenStax). Copyright 2022, OpenStax College, published by Rice University.

**FIGURE 3:**
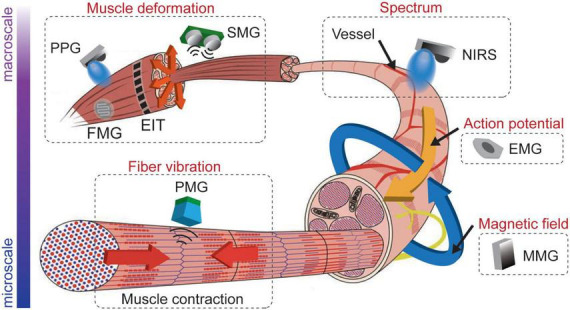
Examples of myography sensing technologies from macroscale to microscale.

Muscle contraction generates detectable biomechanical, biochemical, and bioelectrical signals, as illustrated in Figure 2. The muscle contraction is triggered by forming a cross-bridge between actin and the myosin heads (Figure 2A), resulting in a mechanical change in the muscle. These biomechanical changes bring a tension variation on the skin surface, pushing vessels around the muscle and changing muscle shapes. These mechanical variations enable forcemyography (FMG), phonomyography (PMG), photoplethysmography (PPG), mechanomyography (MeMG, acoustic myography, sound myography, vibromyography), sonomyography (SMG), and electrical impedance tomography (EIT) to interpret muscle activities. In addition, the energy consumption of the muscle fibers during the contraction alters the chemical properties of some biomolecules in blood and muscle. The hemoglobin and myoglobin have different absorption spectra when they carry and lose oxygen, as shown in Figure 2B, which can be detected using the optical method like near-infrared spectroscopy (NIRS). Bioelectrical signals arise in the polarization of a muscle fiber membrane by the neurotransmitter from the nerve-muscle junction, containing action potentials and local currents propagating on the muscle fiber, as illustrated in Figure 2C. Thus, muscle activities can be recorded electrically (EMG) by the sum of these potentials along the muscle fibers (Ahmad, 2012), and magnetically (Magnetomyography, MMG) by the magnetic field generated from the local current (Broser et al., 2018; Zuo et al., 2020a,c). The scale of these myography signal sources lies from macroscale to microscale, resulting in diverse sensing protocols, as demonstrated in Figure 3. Macroscale sensing protocol includes technologies like PPG, FMG, EIT, and SMG, which measure the effects generated by muscle deformation. Signals in macroscale have a large SNR compared to other protocols due to their pronounced variation in spatial scale. However, this prevents them from having a higher temporal resolution, as large-scale spatial variations introduce a certain time delay. Locating deep signal sources from a macroscale sensing protocol like FMG is difficult because the superimposition of superficial and deep muscle deformation is indistinguishable. Although microscale signals might be noisier and harder to detect due to their faint magnitude, they have a larger potential to allocate myography signals with higher spatial resolution since their signal source is more dispersed in the muscle. NIRS uses an optical method to detect microscale signals from vessels inside the muscle in terms of spectrum (Barstow, 2019). Microscale signal measurement for action potentials and magnetic fields that propagate along the muscle fibers are the most promising methods for high temporal and spatial resolution for wearable applications, because they are easy to fabricate with a standard CMOS process (Heidari et al., 2015; Zuo et al., 2019; Zhang et al., 2021). PMG detects the muscle fiber vibration on the microscale, which is adopted in medical diagnosis and as an assistance method for EMG (Orizio et al., 1996; Guo et al., 2017a; Ding et al., 2018). State-of-the-art for these sensing technologies for MMI will be introduced in the following sections regarding different muscle signal forms.

## Biomechanical sensing interfaces

Muscle contraction by the deformation of the muscle fibers will directly bring some mechanical changes, including muscle fiber length, cross-sectional muscle area, muscle shape, surface tension, blood flow velocity, and the positions of vessels around the muscle. Biomechanical sensing interfaces are classified as sensors that interpret muscle information by detecting these mechanical changes. This section investigates and methodically compares the FMG, PMG, SMG, and EIT sensors.

### Forcemyographic sensor

**FIGURE 4:**
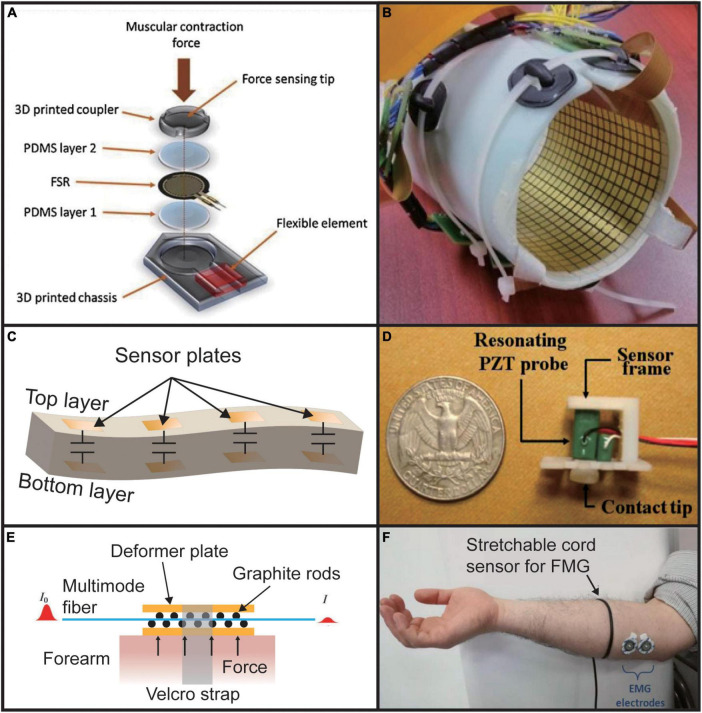
Different types of FMG sensors. **(A)** RPTF-based sensor. Reproduced with permission (Prakash et al., 2021). Copyright 2020, Elsevier; **(B)** high density piezo-resistance sensor array. Reproduced with permission (Radmand et al., 2016). **(C)** Capacitance-based sensor (Truong et al., 2018). **(D)** Piezoelectric-based sensor. Reproduced with permission (Han and Kim, 2013). Copyright 2013, Elsevier. **(E)** Optical fiber-based sensor (Fujiwara et al., 2018). **(F)** Wide range stretch sensor. Adapted from Bifulco et al. (2017). Copyright 2017, IEEE.

Forcemyography is a technology that deciphers the limbs’ movement by sensing the changes in muscle stiffness or the tension formed on the skin surface due to the volumetric changes caused by muscle contraction (Delva et al., 2020). FMG signal acquisition can be achieved through different sensor designs, with some examples given in Figure 4. The common ones are piezo-resistance or resistive polymer-thick-film-based (RPTF-based) sensor (Dordević et al., 2011; Xiao and Menon, 2014; Radmand et al., 2016; Dwivedi et al., 2019; Liang et al., 2019a,b; Barioul et al., 2020; Prakash et al., 2020, 2021; Lei et al., 2021), capacitance-based sensor (Meyer et al., 2006; Truong et al., 2018), piezoelectric-based sensor (Han and Kim, 2013; Farooq and Sazonov, 2017), optical-fiber based sensor (Fujiwara et al., 2018), wide range stretch sensor/conductive rubber (Amft et al., 2006; Bifulco et al., 2017). RPTF-based FMG sensor accounts for more than half of the literature because this kind of sensor has a relatively simple read-out circuit: the core design includes a voltage divider with a buffer, which facilitates its application as a low-cost, high-density array configuration (Xiao and Menon, 2019).

Because FMG senses the volumetric changes of muscle, a relatively low sampling rate is enough. Most researchers set the sampling frequency to 100 Hz, but a study by Lei et al. (2021) shows that 5 Hz is enough for static finger movements. A low sampling rate might mean that FMG might have a limitation in classification latency. Researchers have also shown that increasing the number of FMG recording channels can significantly increase the accuracy of gesture classification (Radmand et al., 2016; Lei et al., 2021). The Force sensing resistor (FSR, Interlink Electronics, Inc, Camarillo, CA, USA) is the most wide-use commercial product of RPTF.

Forcemyography is one of the most readily collected muscle signals, because of its high SNR compared to other muscle signals. Recently, on a low-cost FMG wearable device composed of a pair of FSRs, researchers have achieved an overall success rate in two different gesture recognition sets with six gestures in each group more than 95% (Prakash et al., 2020). A two-layer array with 14 capacitive sensors was also explored for recording FMG, which achieved 95% classification in an experiment with 20 participants and 15 gesture classification accuracy (Truong et al., 2018). Benefiting from their array design, they also verified the algorithm for determining the relative position of the wristband on the arm and found out that the loss of classification performance caused by different wearing positions of the sensor array could be eliminated through such an algorithm. Finally, they achieved an accuracy of 92.4–99.5% in eight location divisions. A High-density FMG (HD-FMG) has been developed for prosthetic control (Radmand et al., 2016). They used an array with 126 recording positions placed on the forearm to acquire the pressure map of the entire arm, which was then classified into eight motions. It achieved an error rate of 0.33%. They also found that the accuracy of classification can be improved by selecting the appropriate location of the FMG sensor array to reduce the influence of external pressure.

Although FMG has a high SNR, it still faces many problems. If a small number of sensors are used, the spatial resolution is low, resulting in being more susceptible to adjacent muscle crosstalk and fatigue (Grushko et al., 2020). At the same time, RPTF, which occupies the leading market position of FMG, has undeniable non-linear characteristics, so its sensor readings cannot be directly correlated with muscle stiffness, which makes precise normalization within an RPTF-based sensor array difficult (Xiao and Menon, 2019). Therefore, it is urgent to develop a more linear and stable sensor that records the pressure signal more consistently to eliminate the effects of differences between users and application scenarios. Another point is that FMG is easily affected by external forces. Even if only a small force is applied, the accuracy of FMG in the classification test will be drastically reduced (Radmand et al., 2016).

### Phonomyographic sensor

**FIGURE 5:**
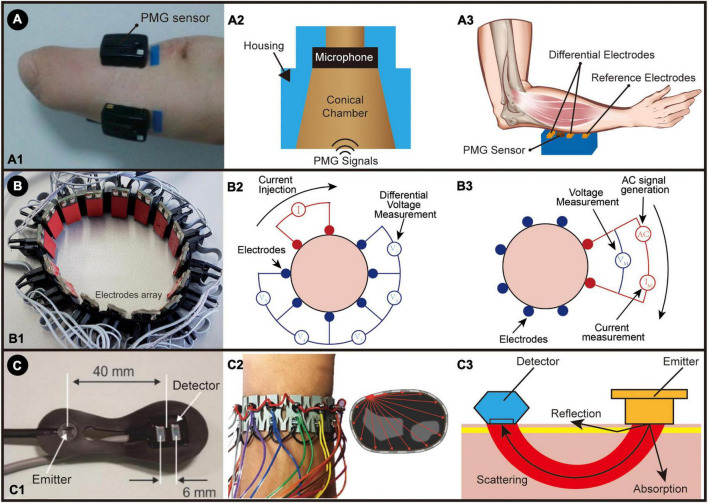
**(A)** The PMG system: **(A1)** The placement of a hybrid EMG-PMG sensor on an amputee. Adapted from Guo et al. (2017a). Copyright 2017, IEEE; **(A2)** An illustration of PMG sensor; **(A3)** An illustration of PMG sensor working with surface electromyography (hybrid EMG-PMG). **(B)** The EIT system: **(B1)** wrist-band setup for EIT system; **(B2)** the illustration of two-terminal configuration measurement; **(B3)** the illustration of four-terminal configuration measurement. **(C)** NIR system: **(C1)** the portable NIRS optic array developed by Everdell et al. (2013) A: light source, B: photodiode detectors. Reproduced with permission (Everdell et al., 2013). Copyright 2013, Elsevier; **(C2)** Operation principle of SensIR: one emitter is on, and all the receivers will capture the scattering light through the tissue, which is repeated for all the emitters. Adapted from McIntosh et al. (2017). Copyright 2017, ACM; **(C3)** illustration of NIR sensor.

Phonomyography (PMG, mechanomyography, MeMG, acoustic myography, sound myography, vibromyography) records low-frequency small vibrations of muscle fibers generated during contractions. The history of modern development and applications of PMG technology began in the 1980s, summarized by Stokes and Blythe (Orizio, 1993; Stokes and Blythe, 2001). Phonomyography and FMG are often easy to confuse. FMG is a more static signal independent of frequency, while PMG signal is wide in the frequency spectrum. One example of the experiment setup for PMG is shown in Figure 5A1 (Guo et al., 2017a).

Many reports have proven that PMG can quantitatively and non-invasively reflect muscle activity (Cochrane-Snyman et al., 2016; Lozano-García et al., 2018; Keller et al., 2019; Mialland et al., 2021). The muscle vibrations first reach a peak due to the profound changes in muscle shape, producing a series of lateral resonances in the muscle fibers (Orizio, 1993). These vibrations with frequencies ranging from 5 to 100 Hz and a displacement amplitude of about 500 nm can be obtained through contact transducers such as microphones, piezoelectric sensors or an accelerometer placed on the skin over the belly of the muscle (Wilson and Vaidyanathan, 2017; Esposito et al., 2018; Keller et al., 2019). The structure of the PMG sensor using a microphone is shown in Figure 5A2. To properly acquire the PMG signal, the sampling frequency should be set to higher than 200 Hz, according to the Nyquist–Shannon sampling theorem.

Phonomyography possesses several advantages over other muscle sensors as a control input source for active powered prostheses. PMG is relatively easy to set up. Because of their contactless measurement, it is less susceptible to changes in skin conditions (such as skin quality and sweating). However, artifacts caused by the movement of the sensor and the noise of the surrounding environment bring significant challenges to its accuracy (Silva et al., 2005; Wilson and Vaidyanathan, 2017). Actually, using a PMG sensor alone is not enough to control a dexterous robotic arm, considering its relatively low accuracy. Still, when combined with EMG, higher overall accuracy can be achieved because EMG and PMG do not interfere with each other. A hybrid EMG-PMG system is illustrated in Figure 5A3. This hybrid method works as a multimodal input source that can provide supplementary information about muscle activity (Silva et al., 2005; Guo et al., 2017a; Grushko et al., 2020). The SNR can also be improved using a pair of accelerometer-microphone sensors (Silva and Chau, 2003). In summary, using PMG alone as an MMI is not feasible at this stage and requires the combination of PMG with other MMI sensors to achieve an effective level of control. In sensor fusion, PMG is used more as a secondary technology to enhance the accuracy of other sensors.

### Sonomyographic sensor

**FIGURE 6:**
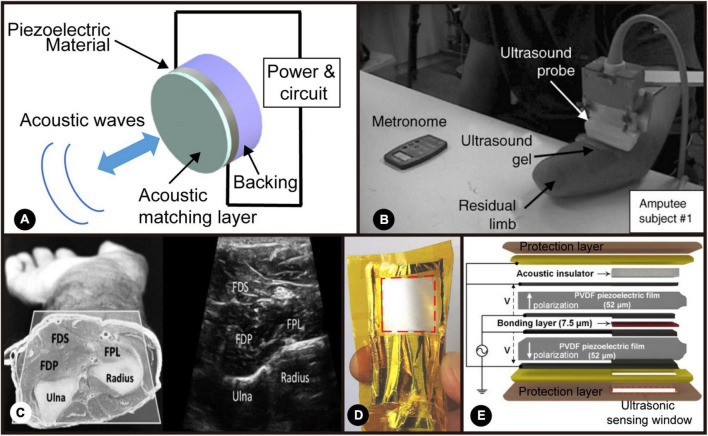
**(A)** Schematic of an ultrasonic transducer. Adapted from Sanchez et al. (2021). Copyright 2021, IEEE; **(B)** Experimental set-up by Zheng et al. (2006). Reproduced with permission (Zheng et al., 2006). Copyright 2006, Elsevier; **(C)** the cross-section and ultrasound image of the forearm muscles by Akhlaghi et al. (2016). Adapted from Akhlaghi et al. (2016). Copyright 2016, IEEE; **(D)** the photo of wearable ultrasonic sensor developed by AlMohimeed and Ono (2020). Reproduced with permission (AlMohimeed and Ono, 2020). Copyright 2006, MDPI; **(E)** schematic of the flexible, single-element ultrasonic transducer by AlMohimeed and Ono (2020). Reproduced with permission (AlMohimeed and Ono, 2020). Copyright 2006, MDPI.

Sonomyography is a relatively recent technique in studying muscle anatomy and physiology that utilizes ultrasonic transducers to measure muscle activity. The method as a whole is quite simple since it utilizes the principles of ultrasound, the pulse-echo phenomenon, and the piezoelectric effect to create an ultrasonic wavefront that travels from the transducer to the muscle, which is then reflected, sensed and further processed by the same device. The critical elements of any transducer or ultrasonic sensor are its backing, piezoelectric material, and acoustic matching layer, as demonstrated in Figure 6A.

Sonomyography was first presented in Zheng et al. (2006) where the main objective was to replace EMG-controlled prostheses with SMG-controlled ones, which is shown in Figure 6B. An ultrasound image of this type of experimental setup is also presented in Figure 6C. Within this study, some of the main challenges of using EMG were highlighted, such as the fact that it is sometimes an invasive technique, mainly when using needle electrodes, its inadequacy at the time of distinguishing the distinct movements of different muscle groups and its inability to gather signals from deep-in-the-body muscles. This is where the authors hypothesized SMG could be an alternative.

Within the last decade, more research has been conducted on SMG for various applications, and it has been shown to have great potential in studying muscle structure and function. Studies have shown this technique provides robust signals with high specificity and better penetration depth than EMG and sub-millimeter spatial resolution, whilst still being non-invasive (Seo et al., 2016; Yang et al., 2018; Akhlaghi et al., 2020). In addition, research has also shown the potential of SMG as a complementary tool to more established methods such as surface EMG (sEMG). Studies have demonstrated its ability to detect with significant precision structural changes within the muscular architecture when maximal voluntary force is exerted. Combining this information with the one obtained from other biomedical signals/images can better assess muscle fatigue (Shi et al., 2007a).

In recent years, SMG is becoming more widely used within the rehabilitation engineering sector because it provides accurate, quantitative information relating to structural changes in the muscles. Various research groups have utilized this technique to study the difference between electrically induced muscle contractions versus voluntary contractions (Qiu et al., 2017; Sheng et al., 2021). This can be used to understand better how different muscular contractions of paralysis patients are to those who are healthy, meaning that their rehabilitation process could potentially be optimized to obtain more similar contractions to those of healthy patients. Furthermore, SMG also offers the advantage of overlooking electrical noise, unlike EMG sensors, meaning that there would be no crosstalk between stimulation impulses created by Functional Electrical Stimulation (FES) and the output generated by the contractions (Grushko et al., 2020).

The use of SMG as a muscle thickness analysis tool is typically the most common within the biomedical imaging sector. Notwithstanding, other parameters can be derived from the muscle thickness, which demonstrates the ability of SMG to provide diverse information about the muscular structure and function. One example of a quantitative parameter could be the wrist angle. There have been studies that have shown how to derive the wrist angle from the changes in the thickness of the forearm muscles (in particular, the extensor carpi radialis) (Shi et al., 2007b; Xie et al., 2009; Guo et al., 2013). Such information could be of great value since it could be used for MMI. Studies have shown that the information obtained from real-time SMG can go through a classification algorithm to determine the different grips the user wants their prosthesis to execute, resulting in an accuracy percentage of 93% over eight trials from five test subjects each (Sikdar et al., 2014; Akhlaghi et al., 2016; Bimbraw et al., 2020). This information can also be applied to assistive MMI that can aid in the rehabilitation process of patients through the creation of interactive and engaging therapies (Akhlaghi et al., 2020; Yang et al., 2020). A flexible and wearable ultrasonic sensor for detecting muscle contractile that was developed by Al-Mohimeed has been shown in Figures 6D,E.

To add to this, research groups like Wang C. et al. (2018) have taken the ultrasonic approach and combined it with stretchable, ultrathin materials to monitor in a non-invasive manner central blood pressure, demonstrating the adaptability and potential of wearable ultrasonic devices.

It is, therefore, undeniable that SMG has great potential, not only in terms of diagnosis of myopathies and peripheral nervous system damage but also as a complementary tool for assistive/rehabilitation engineering, where it can aid patients suffering from paralysis or amputation using techniques ranging from MMI.

### Electrical impedance tomography

Electrical impedance tomography measures the impedance distribution inside the object in real time by measuring the impedance between a set of electrodes placed on the object’s surface. The measurement configuration and an example of EIT are presented in Figure 5B. EIT was first proposed by Henderson and Webster (1978). It was then widely used in medical diagnosis because of its non-invasive, non-radiation, and low-cost characteristics (S Holder, 2004; Ma et al., 2020). Body movements will cause deformations of muscles, and thus the impedance distribution inside the body will also change. The body movements can be inferred by interpreting the changes in EIT images. There are many measurement methods for EIT, the most common of which are two-terminal and four-terminal schemes, as shown in Figures 5B_**2**_, B_**3**_ (Ma et al., 2020). The Two-terminal scheme uses the Volt-ampere method to measure impedance, and only one pair of electrodes is required for each measurement. This method is relatively simple, but the measurement will significantly affect the electrode contact surface, so a larger electrode is usually used to increase the contact area. The Four-terminal scheme uses Kelvin Four-terminal sensing. In this method, a pair of adjacent electrodes is used for AC excitation and current measurement for each measurement, and then the impedance is calculated by measuring the voltage between the remaining electrode pairs. The Kelvin four-wire test can make more accurate measurements. Its key advantage is that the separated current and voltage electrodes eliminate the impedance of wiring and probe contact resistance, making this method less sensitive to changes in skin conditions (Grushko et al., 2020).

The first trial to use EIT for MMI was by Zhang and Harrison (2015). They proposed an EIT system that can be worn on the wrist, called Tomo. The first generation of Tomo integrates eight electrodes and uses the two-terminal scheme measurement method, achieving 96.6% accuracy in 11 gesture recognition tasks. Later, they upgraded Tomo by increasing the number of electrodes and using a four-terminal scheme measurement (Zhang et al., 2016). Wu et al. (2018) optimized the design of the measurement system, using four-terminal schemes so that a minimum of 6.4 Ohm impedance change can be detected, and this system achieved 98% accuracy in 19 gesture classification tasks. EIT can also be used for force detection. Zheng et al. (2019) achieved continuous force measurement through EIT and sigmoid regression with 16 electrodes. Two EITs can be combined to form a 3D EIT system (Yang et al., 2018). The experiment of Jiang et al. proves that the 3D EIT system can more accurately classify certain gestures that are difficult to distinguish by a single-layer EIT system and show better clustering ability (Jiang et al., 2020).

One of the challenges faced by EIT is that as the number of electrodes increases, the time required to reconstruct the impedance distribution will also increase, which inevitably brings a delay. Ma et al. (2020) proposed a new drive pattern that uses fewer electrodes, which reduces the measurement time by 60% without a significant drop in accuracy. Another challenge is that everyone’s baseline conductivity differs, and each person needs to be tuned individually before measuring. Otherwise, it will affect the quality of the EIT images (Zheng et al., 2019).

Another technology similar to EIT is called Capacitance Sensing. This technology is also realized by a set of electrodes placed on the surface of the skin. However, unlike EIT, the electrodes of Capacitance Sensing need to be insulated. A set of fixed emitter electrodes in the array are used to generate an excitation signal and pass the remaining electrodes to measure the entire signal and obtain the whole body’s capacitance in different directions. Muscle contraction will change the distance between the electrodes and the conductivity of the biological tissue between the electrodes, so the capacitance between the electrodes will also vary accordingly. Different muscle contractions will also cause various capacitance changes in the array. The advantage of this technique over EIT is that Capacitance Sensing does not require a lot of computing power to calculate the capacitance distribution of the entire body section, so the recognition delay may be relatively low. At the same time, Capacitance Sensing is not sensitive to the contact between the electrode and the skin because it measures opened gaps. However, sweat will still affect its accuracy because sweat will change the conductivity between the electrodes (Grushko et al., 2020).

It should be noted that the absolute value of the capacitance will change due to the relative movement between the sensor array and the body. Therefore, in actual research, the accuracy of Capacitance Sensing classification is not very high. In the study of Cheng et al. (2012, 2013), a classification accuracy of 58% was achieved for 36 sports modes. The accuracy of capacitance may be achieved by increasing the number of electrodes (Grushko et al., 2020).

## Biochemical sensing interfaces

Muscle contraction brings not only physical changes but also biochemical changes: muscle activities will change the local oxygen concentration in the blood, causing structural changes in hemoglobin and myoglobin, and thus change the scattering of the muscles and vessels of certain light waves. Information on muscle activities could also be interpreted by detecting these biochemical changes using optical methods. The most common technology in the literature is NIRS, based on optoelectronic devices (Bianchi et al., 1999; Chianura and Giardini, 2010; Fukui et al., 2011; Herrmann et al., 2012; Everdell et al., 2013; Muhammed and Raghavendra, 2015; Gong et al., 2016; Guo et al., 2016; McIntosh et al., 2017; Paleari et al., 2017; Wang H. et al., 2019; Nsugbe et al., 2020; Nsugbe, 2021a). Some examples of NIRS are shown in Figure 5C. Human tissues are basically transparent under near-infrared light from 700 to 1,000 nm, and the main chromosomes absorbed in skeletal muscle are hemoglobin and myoglobin (Villringer and Chance, 1997). Depending on whether oxygen is combined, the absorption rate at near-infrared light of hemoglobin and myoglobin differs, commonly referred to as hemodynamics (Nsugbe, 2021a). As shown in Figure 5C3, after the near-infrared light generated by the emitter is scattered in the tissue, the hemodynamics could be detected and analyzed to show different muscle activities (Barstow, 2019).

Near-infrared spectroscopy can easily be configured as an array. The most traditional configuration method is that each emitter corresponds to a receiver, which means that each receiver is only responsible for receiving the infrared light emitted by the neighboring and matching emitter. In this operating mode, all emitters will emit light simultaneously, and the information recorded by each receiver will be fed into the classification algorithm simultaneously. Gong et al. (2016) achieved 89% recognition accuracy of eight gestures by placing 12 infrared (IR) sensors near the wrist. A cyclic scanning method is then adopted in the research done by McIntosh et al. (2017), using a wristband around the arm with 14 emitters and receivers, as shown in Figure 5C_**2**_. Only one emitter emits infrared light in each recording. The light projected and reflected in the entire arm is recorded by all 14 receivers simultaneously and then circulates in all emitters. Fourteen times is a complete recording cycle. In this way, each cycle will generate 196 data, and then they send these data into a multilayer-perceptron classifier for learning and classification. They obtained a 93% accuracy rate in the classification test of 12 gestures.

Literature shows that the classification accuracy of sEMG can be improved when combined with NIRS (Attenberger and Buchenrieder, 2012; Guo et al., 2017b; Paleari et al., 2017; Scano et al., 2019; Nsugbe et al., 2020). Because the distance between the emitter and the detector will determine the depths of IR light scattering, absorption and return to the sensor, there is a potential capability of NIRS to remedy sEMG’s shortcoming in deep muscle detection (Paleari et al., 2017). At the same time, NIRS may also solve the problem of a decrease in the classification accuracy of EMG due to adjacent muscle signal interference and electronic interference (Guo et al., 2017b; Paleari et al., 2017; Nsugbe et al., 2020). Using sEMG and NIRS in combination, an accuracy rate of 92.2% was achieved in the experiment to determine the state of the three hands, far exceeding the accuracy rate of 73.3% when using EMG alone (Guo et al., 2017b). Experiments on three amputees also proved that the combination of NIRS and EMG improved the accuracy by about 15% in the classification test of 10 gestures compared with EMG-only. However, the tricky point is that by changing the distance between the emitter and the sensor, one can control the depth of detection, the most effective detection range is still limited to the superficial muscles, limiting their ability to interpret deep muscle information. Other biological tissues between the sensor and the muscle may also affect NIRS (such as skin with different pigments). At the same time, the deformation of the muscles during contraction may also change the light scattering (Barstow, 2019).

It is worth noting that there is another muscle interface based on optoelectronic sensors, Photoplethysmography (PPG) or some called Optomyography (OMG) (Muhammed and Raghavendra, 2015; Brennan, 2020; Zhao et al., 2021). PPG uses a different way to interpret the effects of muscle activity on photoelectric signals. This technology does not detect changes in chromophores in human tissues but detects the relative displacement of blood vessels and blood flow. Especially in terms of the muscle activity of the hand, the flexor digitorum superficialis and flexor hallucis longus, which control the hand activity, are located just beside the radial artery and the ulnar artery. The muscle activity will squeeze the blood vessels, causing vessel displacement and blood flow changes, and thus hand movements can be reflected in PPG. PPG detects the mechanical changes produced by muscles. We put it in this chapter to better compare the different interfaces based on photoelectric systems. The technology is still in its early stage, and related research is few. Zhao et al. (2021) used two PPG sensors combined with a series of algorithms to remove pulsation and body movement interference and achieved an average accuracy of 88.32% for nine postures. In addition, researchers proposed a sensor that measures facial muscle deformation by measuring changes in the light path (Tamee et al., 2013). In this research, three sensors are combined and attached to the subject’s forehead for muscle diagnosis or control auxiliary equipment, but the system lacks relevant experiments to prove its feasibility. Apple Inc. also uses this technology to control its wristband system, but the experience remains to be seen (Brennan, 2020).

One obvious advantage of NIRS/PPG is that there are many wristband products embedded with optoelectronic sensors on the market. Although further research is needed, these photoelectric sensor-based devices have great potential to become the widely used MMI. Nevertheless, ambient light easily affects optoelectronic devices, so good contact between the sensor and the skin is required to block external light (Grushko et al., 2020).

## Bioelectrical sensing interfaces

Neurotransmitters released from the nerve-muscle junction depolarize the muscle fibers and trigger contraction. During this process, the potential on both sides of the membrane of the muscle fiber changes, while a local current is generated. In this section, we will describe the EMG, which detects the change in potential, and the MMG, which detects the magnetic field generated from the local currents.

### Electromyographic sensor

Electromyography is a method to record the electrical activity of muscles (Reaz et al., 2006). The history of the EMG signals dates back to the 1660s when researchers noticed that the muscles of an electric ray fish could generate electricity (Basmajian and de Luca, 1985). Researchers document the correlation between muscle contraction and electricity in the following 200 years. Nevertheless, it was not until 1890 that the first recording of the electrical activity in a muscle was made by Marey, who introduced the term electromyography (Clarys, 1994). In the 1960s, the EMG signals received more attention, mainly due to the improvements in the recording systems, and were adopted for clinical applications, e.g., in the diagnosis of Huntington’s disease, myopathies and muscular dystrophies (Merletti and Farina, 2009; Narayan et al., 2015; Sadikoglu et al., 2017; Vomero et al., 2018; Rodríguez-Tapia et al., 2020).

Each muscle is innervated by a various number of motor neurons, and every neuron supplies a wide range of muscle fibers in a muscle depending on its function nature. Each motor neuron and the muscle fibers are innervated by the axon of the motor neuron called the motor unit. The number of muscle fibers innervated by a motor neuron in a particular motor unit indicates how fine or precise the motor unit’s movement is. The excitation signal of a motor neuron is approximately equivalent to a digital signal, and the signal generated by the activated muscle fibers is called a phase signal. Motor neurons have different firing frequencies and different peak characteristics of the signal. Thus, when muscle contracts, multiple digital signals with different frequencies will excite the muscle fibers to produce multiple phase signals, and when the endogenous/exogenous noise is included, the EMG signal is obtained. Therefore, the macroscopic EMG signal is essentially an interfering signal containing noise.

**FIGURE 7:**
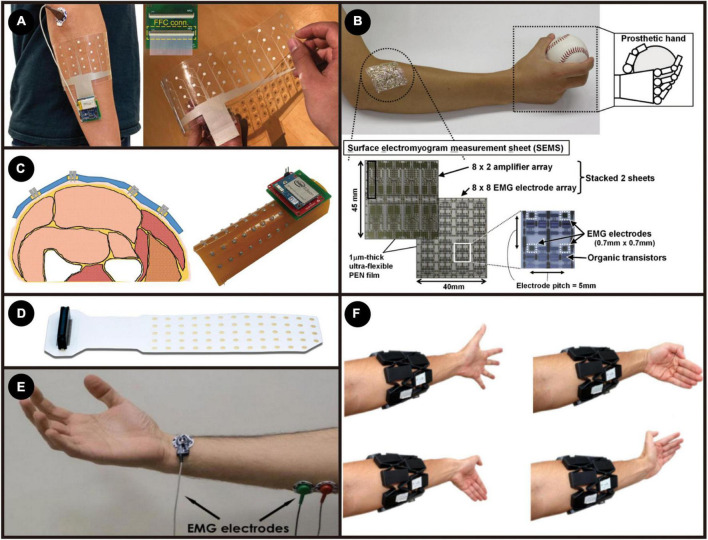
**(A)** The sEMG electrode array and its experiment setup. Adapted from Moin et al. (2021). Copyright 2021, Nature; **(B)** ultra-flexible high-density EMG measurement sheet. Adapted from Fuketa et al. (2014). Copyright 2014, IEEE; **(C)** Microneedle-Based High-Density Electrode Array. Reproduced with permission (Kim et al., 2018). Copyright 2018, Sensors; **(D)** adhesive EMG electrode matrices (GR08MM1305, OT Bioelettronica). **(E)** Bipolar electrode recording setup. Reproduced with permission (Esposito et al., 2018). Copyright 2018, Sensors; **(F)** gesture classification using MyoBand. Reproduced with permission (Simão et al., 2019). Copyright 2019, Elsevier.

Electromyography can be divided into surface EMG (sEMG) and invasive EMG (iEMG). sEMG is the most widely used EMG recording system, especially within the academic sector. It requires adhesive or dry electrodes to be attached to the surface of the skin to pick up the electrical activity of muscles (Drost et al., 2006). The sEMG signal is the algebraic summation of volume conducted MUAPTs from different muscle fibers (Rutkove, 2007). sEMG does not require surgical implantation, so it is easy to wear and replace, which is more acceptable for ordinary people in daily life. So, it has received great attention from academia and industry, and Figure 7 presents examples of existing sEMG technologies.

There are still some problems to be solved while using sEMG. Motion artifacts caused by accidental arm or hand movements or external vibrations, causing extra unwanted signals, limit the application of sEMG in more precise and smooth control (Bi et al., 2019). Usually, chronic recording using sEMG requires frequent calibrations to ensure recorded data consistency. The electrolyte and bio-fluid produced by perspiration accumulated under the recording sites will change the skin-electrode interface’s impedance, thus influencing the signal characteristics (Muñoz et al., 2002; Hargrove et al., 2007). This variation might bring problems when decoding sEMG into MUAPTs. Another significant disadvantage of sEMG is that signals for deeper or smaller muscles are hard to detect from the skin’s surface. That is because MUAPTs need to penetrate different layers of the body tissues to reach the recording sites, causing unwanted blurring effects and degradation, making it challenging to differentiate the source of the signal generated in a muscle. The blurring effect causes relatively poor spatial resolution, especially for large-size electrodes (Lewis et al., 2013; Afsharipour et al., 2019). All these drawbacks limit the development of sEMG as a more accurate, effective, and smooth MMI or diagnostic tool.

Recently, some dry electrodes made with materials with modulus, thickness, and other physical properties well-matched to the skin have also been used to record sEMG (Kim et al., 2011, 2016; Liu et al., 2016; Nawrocki et al., 2018). These devices only need to use Van der Waals force to firmly adhere to the skin without any mechanical fixation or glue, which can effectively reduce the impact of motion artifacts (Park et al., 2015; Wang Y. et al., 2018). Although such electrodes can be reused, these novel electrodes’ long-term stability still needs to be investigated (Kim et al., 2016).

For sEMG, works have shown that a higher overall accuracy and less latency of the control process could be achieved by increasing the number of electrode sites to form a high-density sensor array (Muceli and Farina, 2012; Amma et al., 2015; Geng et al., 2016; Tam et al., 2020; Holobar and Farina, 2021). A smaller number of sEMG systems will be more likely to rely on frequency or time features and thus have limitations on the sampling frequency, processing time windows, and consequently higher latency. By having more sEMG electrodes to cover a larger sampling area and thus obtain the spatial domain features, less reliance on the time or frequency domain ultimately improves the latency of the whole system (Xu et al., 2016; Moin et al., 2021; Tam et al., 2021).

Because the EMG signal is relatively weak and contains much noise, the read-out circuit needs to be carefully designed. While appropriately amplifying the EMG signal, it is necessary to remove the noise that comes with the signal and introduce as little noise as possible on the line. The read-out circuits for both surface and implantable EMG systems usually consist of amplifiers, filters, and analog-digital converters (ADCs). The maximum amplitude of the EMG signal is around 10 mVpp, and the minimum is only 14 uVpp (Farnsworth et al., 2008). For most bio-signal ADCs, the resolution is a few millivolts. Therefore, an amplifier stage is needed to properly amplify the EMG signal to make the best use of the ADC’s output bits so that the signal after the analog-to-digital conversion will have less distortion. The main component of the EMG spectrum is between 40 and 600 Hz (Ruff et al., 2010; Ng et al., 2020). When measuring EMG signals, there will be some other unwanted interference signals. For example, the movement artifacts between 1 and 3 Hz and the 50 Hz Mains-hum from the power line (Ruff et al., 2010). Therefore, there is usually a filtering stage to filter out unwanted frequencies to reduce the ADC jitter after amplification. For on-chip integrated multi-channel recording systems, analog multiplexers are also essential (Han et al., 2013). The integrated circuit of the ADC is more complex than amplifiers and filters, so if each channel is equipped with an ADC, it will take up much area on the chip, and the power consumption will also increase significantly. For a wearable EMG system that integrates the electrodes with the read-out circuit and other devices, the design lies in designing a signal processing circuit with a more compact size, lower noise, and lower energy consumption (Mastinu et al., 2015; Ng et al., 2020).

### Magnetomyographic sensor

With the rapid development of micro- and nanoscale magnetic sensors, non-invasive recording of the magnetic manifestation of muscle activity has become a reliable and robust approach for biomedical applications since it has great potential to improve medical diagnosis and health monitoring, and to develop assistive/rehabilitation robotics where the HMI can assist the disabled with limb difference to perform essential activities of daily living (Zuo et al., 2020a). Detecting weak magnetic signals derived from human skeletal muscle, was first formally proposed in 1972 and it was called MMG(Cohen and Givler, 1972). Scientists have recorded the magnetomyogram signal as one component of the magnetic field vector for the time at the point of measurement, in which the magnetic fields are by cause of currents produced from the skeletal muscle (Cohen and Givler, 1972; Masuda et al., 1999; Wijesinghe, 2014; Barbieri et al., 2016a,b). Compared to a well-established EMG technique, the MMG measurement has become an effective alternative way due to its significantly higher spatial resolution despite the same temporal resolution as the EMG signals. In addition, the non-invasive MMG offers vector information of the muscle movement, long-term biocompatibility with tissue, a higher signal-to-noise, and better positioning and fast screening of sensors without electric contacts (Zuo et al., 2020a).

**FIGURE 8:**
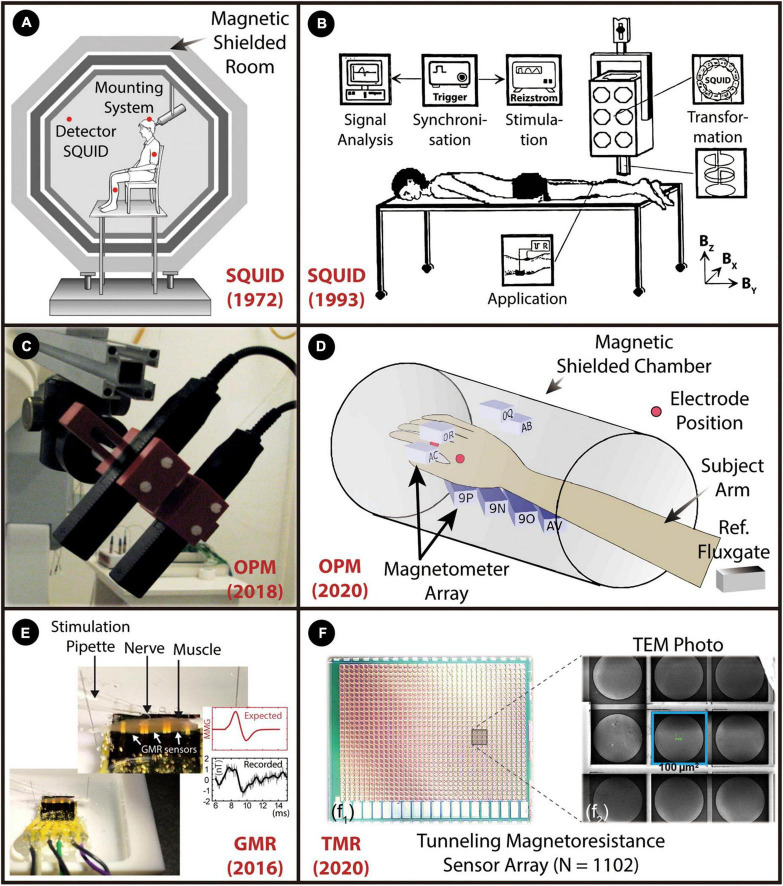
A graphical overview of the weak MMG detection from skeletal muscle. The figure shows the miniaturization pathway from bulky SQUIDs to spintronic nanoscale sensors. **(A)** SQUID magnetometer. Reproduced with permission (Cohen and Givler, 1972). Copyright 1972, Elsevier **(B)**, Room SQUID system. Reproduced with permission (Reincke, 1993). Copyright 1993, Biomed Tech (Berlin), **(C)** OPM based on the zero-field resonance of spin-polarized rubidium atoms in. Reproduced with permission (Broser et al., 2018). Copyright 2018, IEEE, **(D)** OPM array. Reproduced with permission. **(E)** Spintronics sensor based on GMR effect. Reproduced with permission (Barbieri et al., 2016b). Copyright 2016, Springer Nature, **(F)** TMR sensor array with 1102 TMR elements are connected in 38 series and 29 parallel. Reproduced with permission (Zuo et al., 2020b). Copyright 2020, IEEE.

The magnitude of EMG signals is on the scale of milli-volts. However, the MMG signal is in the range of femto (10^–15^) to pico (10^–12^) Tesla, inversely proportional to the distance between the measurement point and the skeletal muscle (Garcia and Baffa, 2015). The vision of using the principles of magnetism to overcome the challenges of recording electrical signals from the peripheral muscle system is building up incredible momentum. In 1972, Cohen and Givler discovered the MMG signals using superconducting quantum interference devices (SQUIDs) performed in a large magnetically shielded room, as shown in Figure 8A (Cohen and Givler, 1972). Later on, Reincke investigated the neuromuscular system in humans using a SQUID magnetometer with a second-order gradiometer detector, as illustrated in Figure 8B (Reincke, 1993). However, the ultra-high cost of the devices and the complexity of the setup, requiring a temperature-controlled environment with the removal of the magnetic background noise, limit the spread of this sensing technique. Multiple magnetic sensing techniques have been widely explored over the past years as an effective alternative pico-Tesla biosensing approach at room temperature (Zuo et al., 2020a). Recently, optically-pumped magnetometers (OPM), from competing manufacturers, e.g., QuSpin Inc., FieldLine Inc. and Twinleaf with a below 100 fT/√Hz sensitivity (Figure 8C) (Alem et al., 2015; Boto et al., 2017), were implemented to record evoked MMG signals to study the innervation of the nerves in hand muscles of human subjects (Broser et al., 2018; Elzenheimer et al., 2020). It has been utilized to analyze the signal conduction in muscular fibers and the spatio-temporal dynamics of the magnetic field generated by the propagating muscle action potential. Unfortunately, current OPM technology mandates heating the sensor, resulting in surface temperatures of around 40°C, and requires the background magnetic field to be below ∼50 nT – well below the Earth’s magnetic field and typical noise sources (line noise, equipment noise, elevators, cars, etc.). As shown in Figure 8D, the recent achievement in a regular hospital examination room was by using a portable magnetic shield that only encompasses the arm of the subject.

Magnetic sensors based on the spintronics effect have been widely explored over the past years as an effective alternative pico-Tesla biosensing approach at room temperature. Spintronics studies a fundamental property of electrons known as their spin. Some materials exhibit spin-related magneto-resistive properties at room temperature. That is, their electrical resistance is a function of the magnitude and the direction of the applied magnetic field. This phenomenon has led to the development of a spintronic sensor that can detect pico-Tesla level magnetic fields, appropriate for MMG sensing. The physical size of a typical spintronic sensor is significantly smaller than that of a SQUID or an OPM. Recently, giant magnetoresistance (GMR) sensors were used to record the MMG signal from the surface of a muscle in mice (Barbieri et al., 2016b), as demonstrated in Figure 8E. However, the sensitivity of GMR sensors is in the nano-Tesla range, and thus averaging was required to enhance the SNR. Recent developments in physics and materials promise a new class of solid-state spintronic sensors based on the tunnel magnetoresistive (TMR) effect to sense pico-Tesla/√Hz magnetic field (Freitas et al., 2007, 2016), which are faster, more reliable and of lower power than the existing spintronic sensors. A recent study is shown that, for the first time, identification, characterization, and quantification of the MMG signals at room temperature through an ultra-miniaturized and highly sensitive TMR sensor array (1,102 elements), as shown in Figure 8F_**1**_ (Zuo et al., 2020b). An enlarged image with a size of 100 × 100 μm per TMR element is illustrated in Figure 8F_**2**_. The sensor array was precisely placed on the hand skin of the abductor pollicis brevis muscle to record the lateral component of the magnetic signal at room temperature. Further development of this technique is highly dependent on isolating the weak biomagnetic signals from background noise and canceling the geomagnetic field in real-time. In addition, to avoid the effects of movements as much as possible, implantable MMG sensors would be more appropriate for HMIs, such as the control of prosthetic limbs, to reduce the effect of muscle movement.

## Discussion and outlook

As reported above, various myography sensing technologies for wearable MMIs have been developed and investigated by interpreting different muscle signal forms during the past decades. This creates more convenient and comfortable applications for healthcare, assistive/rehabilitation robotics, and human augmentation. However, there are still challenges that need to be conquered to achieve more natural and accurate measurements. Super-resolution provides a novel benchmark to design the hardware of myography, opening a realm of applications in which wearable MMI sensors can offer advantages. The super-resolution is reflected in two aspects: (a) a high-density sensor array for collecting clear signals over a large area and (b) the ability to detect signals from a certain depth or distance. In the following, we present a cross-sectional comparison of the vulnerability and the detectability of the mentioned wearable MMI sensors as super-resolution myography. We then briefly investigate the continuous model and the fusion of different sensors as the future development with super-resolution.

### Vulnerability

**FIGURE 9:**
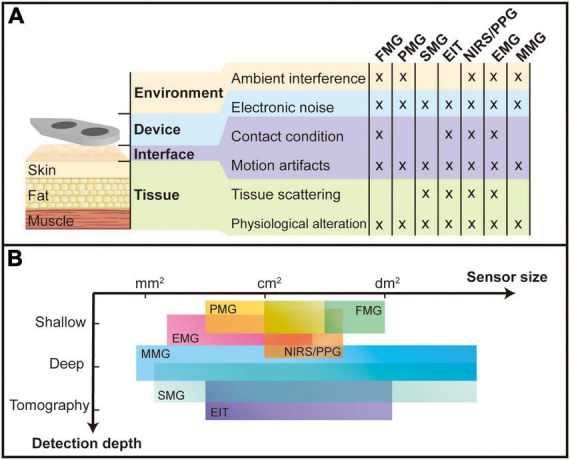
Comparison of different myography sensor. **(A)** Types of interference to which various sensors are subjected; **(B)** the sensor size and detection depth for different myography sensors. The unit “mm^2^,” “cm^2^,” and “dm^2^” stand for squared millimeter (10^– 6^ m^2^), squared centimeter (10^– 4^ m^2^), and squared decimeter (10^– 2^ m^2^), respectively.

Stable and repeatable recording of the muscle signal plays a key role in MMI applications. For wearable devices, noise and interference introduce huge variations and distortion on the path of signal from the source to the sensor, which comes from four places: tissue, interface, device, and environment. The scattering effects and properties altering in the biological tissue bring distortions and changes to signal. The condition modification and relative displacement of the interface between the sensor and the biological tissue cause drift and inconsistency of the signal. Noise introduced by the sensor and its readout circuit hinder a higher sensor detection limit. The interference from other identical sources in the environment may drown out the weak biosignals from the muscle and introduce a large DC component. These factors of vulnerability are summarized from the previous sections and compared in Figure 9A.

In the comparison, it is noted that all devices are affected by electronic noise, motion artifacts, and physiological alteration, probably because these factors are on the obligatory path of all signal forms. Physiological alteration includes muscle fatigue and the loss or increase of muscles, fat, or any other tissue between the sensor and the source muscle. These alterations change the transmission properties and thus influence the signal quality. Motion artifacts mean changes in the relative position of the sensor and the muscle, resulting in a different transmission path of the signal from source to sensor. The high-density sensor array used in the super-resolution design promises to solve this problem: by mapping a full muscle scale signal, the shifting of any device can be monitored by a designed algorithm, which keeps track of the shifting of a reference pattern (Xiao and Menon, 2019). Electronic noise includes thermal noise, flicker noise, and power line noise in the sensor system. Electronic noise has different influences on different MMI sensors. Sensors measuring biomechanical signals are relatively less affected as these signals tend to be more pronounced and thus have a relatively higher SNR. External forces, sounds (vibrations), light, electrical potentials from stimulation and magnetic fields from the earth and other surrounding devices are ambient interference for FMG, PMG, NIRS/PPG, EMG, and MMG, respectively, which largely affects the practical applications of MMI. The contact condition also influences sensing technologies like FMG, EIT, NIRS/PPG, and EMG because they require direct contact with the tissue to measure the signal. Vibration, electrical fields, and light signals will be scattered inside the tissue and result in the blurring of signals, which is a challenge for these sensing technologies (SMG, EIT, NIRS/PPG, EMG) to have higher resolution in depth.

Among all the sensors, although EMG sensors are affected by all the interference, they are still commonly used with good performance due to their low-cost and relatively simple setup for a high-density array to compensate for various disturbances. The recent advances on the remaining sensors with fewer interference terms could offer good prospects and provide more accurate control than EMG.

### Detectability

Spatial resolution, defined as the capability of distinguishing adjacent muscle signals, is one of the criteria for detectability as super-resolution myography. Different muscles play different roles during limb movement, and accurate identification of the activity patterns of different muscles can help to understand the antagonism between muscles. It is shown that further miniaturizing the sensor and increasing the density and channel number into an array form can increase the system’s spatial resolution and overall accuracy due to more features in the recorded signal (Tawil et al., 2011; Muceli and Farina, 2012; Radmand et al., 2016; Reermann et al., 2019; Grushko et al., 2020; Lei et al., 2021). The comparison is made in Figure 9B, showing the size level of different sensing units. Sensing technologies like EMG, MMG, and SMG that detect microscale changes during muscle activities could achieve a smaller sensing unit and thus might have the potential to possess higher spatial resolution. On the one hand, the combination of algorithms and electrode arrays can better adapt to signal variations due to sweating, muscle strain, sensor misalignment, etc. (Amma et al., 2015; Truong et al., 2018; Moin et al., 2021). Studies have shown that the high-density EMG technique can effectively counteract the problem of signals from some small muscle groups being masked due to crosstalk (Muceli et al., 2015; Radmand et al., 2016; Wei et al., 2019). On the other hand, the higher spatial resolution results in more local properties picked up. If a small number of sensing units are used to record muscle activity in a limited area, the final measurement may be over-pronounced by the activity locally. The high spatial selectivity might also bring the problem that it is easily affected by motion artifacts and the offset between the sensor and recording location (Klotz et al., 2022).

Non-invasive deep muscle detection is another metric for super-resolution myography, allowing more deep muscle activity to be recorded without surgery and improving the accuracy of interpretation (He et al., 2019; Yang et al., 2020). The three levels of deep muscle detectability have been assigned to every MMI sensor: shallow, deep and tomography, as shown in Figure 9B. Due to a blurring effect and volume conduction, EMG, PMG and FMG can only detect shallow muscles, while NIRS/PPG could have different detection depths by changing the distance between the emitter and the receiver. However, as mentioned in section “Bioelectrical sensing interfaces,” the most effective detection depth of NIRS/PPG remains on the surface of the muscle. MMG has great potential to detect deep muscle contractions as all body tissues are magnetically transparent, and the magnetic fields propagate to the surface without distortion. Nevertheless, further research needs to be carried out in this research field, solving magnetic interference problems from the surrounding environment. SMG and EIT could detect deep muscles and even output the tomography of the detection area. However, they are limited in real-time applications by a relatively significant delay due to the complex process of raw signals.

Table 1 summarizes the channel number, channel density, classification performance, and power consumption for recent sensing technologies. The channel density is acquired by dividing the total channel numbers by the total sensing area. So, this represents how much the number of channels in a unit area could one sensing technology achieve. A higher spatial resolution is expected when there is higher channel density. The channel density of a high-density sensor array in literature is normally larger than 0.25 cm^–2^ (like FMG, EMG, and EIT), and the super-resolution sensor array is more than 10 cm^–2^ (like SMG and MMG). Additionally, the number of sensor arrays depends on the number of ADC channels. There are two standard configurations: (1) one ADC for each sensor unit, which is simple but takes up a lot of space and is power-hungry with the high number of channels; (2) one ADC for multiple sensors by sharing a common reference or using a multiplexor for switching sensors, called a line scanning structure (Lei et al., 2021).

**TABLE 1:** Recent typical works of each sensing technology and their performance.

Sensor type	Work	Channel number	Channel density	Number of tasks	Accuracy	Real-time	Latency	Power consumption
**FMG**	Kahanowich and Sintov, 2021	15	∼0.15 cm^–2^	5	97.5%	Yes	0.02 s	Not mentioned
	Ahmadizadeh et al., 2019	16	∼3.3 cm^–2^	6	88.0%	No	–	Not mentioned
	Truong et al., 2018	15	∼0.4 cm^–2^	15	95%	Yes	0.015s	69 μW
	Radmand et al., 2016	126	∼0.9 cm^–2^	8	99.7%	No	–	Not mentioned
	Dementyev and Paradiso, 2014	15	Not known	5	80.5%	Yes	1.6 s	60.7 μW
**PMG**	Al-Timemy et al., 2022	8	Not known	14	88%	No	–	Not mentioned
**SMG**	Bimbraw et al., 2020	1	–	4	93%	Yes	Not mentioned	Not mentioned
	AlMohimeed and Ono, 2020	1	∼5 cm^–2^	–	–	–	–	Not mentioned
	Wang C. et al., 2018	20	∼33 cm^–2^	–	–	–	–	Not mentioned
	Yang et al., 2018	4	Not known	11	95.4%	Yes	0.243 s	6 W
**PPG**	Zhao et al., 2021	2	Not known	9	98%	Yes	0.601 s s	310 mW
**EIT**	Chen et al., 2021	16	Not known	11	98%	Yes	Not mentioned	Not mentioned
	Jiang et al., 2020	16	Not Known	8	99.5%	No	–	1.1 W
	Ma et al., 2020	8	Not known	11	97.5%	No	–	Not mentioned
	Zhang and Harrison, 2015	8	Not known	8	96.6%	Not mentioned	–	50 mW
**NIRS**	Nsugbe, 2021a	14	∼0.1 cm^–2^	4	56%	No	–	Not mentioned
	McIntosh et al., 2017	14	∼0.1 cm^–2^	12	93.3%	No	–	450 mW
	Guo et al., 2017b *	4	Not known	10	85%	Yes	1.62 s	280 mW
**EMG**	Godoy et al., 2022	16	Not know	6	91.66%	Yes	0.00126 s	Not mentioned
	Moin et al., 2021	64	0.27 cm^–2^	21	92.87%	Yes	0.1 s	∼150 mW
	Wu et al., 2021	1	Not known	–	–	–	–	4.735 mW
	Fuketa et al., 2014	16	∼0.9 cm^–2^	–	–	–	–	30 μW
**MMG**	Zuo et al., 2020a	1	∼4.2 cm^–2^	–	–	–	–	Sub-mW

*This work contains a hybrid of NIRS and EMG. The accuracy presented here is obtained under NIRS-only working mode. However, the power consumption is obtained in hybrid EMG-NIRS working mode.

Table 2 compares sensors in terms of size, high-density configuration, and non-invasive deep muscle detectability—which are the three most important parameters of super-resolution. PMG and PPG do not have high-density array implementations yet, which might be due to their relatively large sensor size. EIT is inherent with multiple electrodes. Only three sensing technologies are reported to have deep muscle detectability—SMG, EIT, and MMG.

**TABLE 2:** General comparison between myography sensing technologies.

Sensor type	Signal feature	Typical sensor size	High-density array	Non-invasive deep muscle detection	Portability	Typical power consumption	Typical hybrid pair
FMG	Biomechanical	Small	Yes	No	Yes	Low	sEMG
PMG	Biomechanical	Large	No	No	Yes	Medium	sEMG, NIRS
SMG	Biomechanical	Small	Yes	Yes	Yes	High	–
PPG	Biomechanical	Large	No	No	Yes	High	–
EIT	Biomechanical	Large	No	Yes	Yes	High	–
NIRS	Biochemical	Large	Yes	No	Yes	High	sEMG, PMG
sEMG	Bioelectrical	Medium	Yes	No	Yes	Low	FMG, PMG, NIRS
MMG	Bioelectrical	Small	Yes	Yes	Yes	Low	–

Detectability is also influenced by the position in which the sensor is worn. The location of the signal recording is also debated for recording electrical signals. Some researchers place the sensor near the wrist during signal recording. In this way, the signal loss is less since the muscles’ tendon part is concentrated here, and the biological tissue is relatively thinner (Liang et al., 2019a; Mendez et al., 2021). Instead, other studies suggest that the signal is smaller in the wrist and should be recorded in the muscle belly, the most active part of the muscle (Farina et al., 2014; He et al., 2019; Holobar and Farina, 2021).

### Future of the super-resolution myography

Super-resolution myography sensing has opened a realm of MMI applications with enhanced vulnerability and detectability by utilizing a high-density sensor array for the detection of deep muscle. Future development of super-resolution sensors will open new possibilities for the next generation of myography and make it more practical for versatile applications. In this section, we discuss several potential research themes which could further revitalize super-resolution myography, including continuous models to help in a more natural control process and sensor fusion to help compensate for the detection between different sensing technologies.

#### Continuous model

Most MMI use the pattern recognition approach to decode motion. As a result, different MMI could be compared by the accuracy of classification tasks. Nevertheless, the accuracy of the classification tasks for the current MMI technology is all relatively high (around 90% or higher) and do not offer a significant difference from each other, as shown in Table 1. Since human limb movements are continuous in practical applications, involving multiple degrees of freedom (DOF) of the joints, the parameter space describing these limb movements is often continuous. However, the MMI control set based on pattern classification is limited to a singular decision, and these classifications are only rough discrete approximations of the continuous parameter space. Such discrete approximations can hinder multi-degree-of-freedom control contexts and can also be detrimental to the accuracy of proportional control (Jiang et al., 2012). Support Vector Regression can address this issue to some extent (Ameri et al., 2014; Kadkhodayan et al., 2016). Recently, researchers have successfully validated convolutional neural networks to decode complex wrist movements with three DOFs effectively (Yang et al., 2019). Besides, a study also shows that regression provides a better user correction of control commands than classification (Hahne et al., 2017).

One of the most critical drawbacks in the field of MMI due to the extensive use of classification methods is the lack of real-time evaluation and demonstration (Xu et al., 2016; Tam et al., 2021). Studies typically report accuracy for static classification tasks, and these metrics do not always translate well to dynamic real-time performance (Hahne et al., 2017). We have summarized and compared the typical classification accuracy for different sensing technologies in Table 1 and labeled if this data is acquired under the real-time task with its latency. As can be seen in Table 1, FMG and EMG have good real-time performance in terms of latency. Pattern recognition remains somewhat questionable. An experiment with eight individuals concludes that pattern recognition still offers irreplaceable functional advantages and may also be more suitable for home use in an amputee (Hargrove et al., 2017).

Although we compare the performance of different sensing techniques on classification tasks in Table 1, it is important to note that we should be cautious about comparing the accuracy and general performance. When different protocols, users, and gesture types/numbers are used, pure comparisons of classification tasks are often suspect without proper clinical comparison trials, which are the primary source of bias. These metrics and comparisons can be used as a general frame of reference but cannot be extrapolated from them.

#### Sensor fusion

Sensor fusion is of particular interest as they may be paired to create synergies and compensate for each sensor’s shortcomings. Most studies have implemented EMG as the primary sensor to pair with other different sensors. The typical pairs of sensor fusion are summarized in Table 2. Recent literature shows that combining NIRS improves the classification accuracy of sEMG (Attenberger and Buchenrieder, 2012; Paleari et al., 2017; Nsugbe et al., 2020). Because the distance between the emitter and detector will determine the scattering, absorption and return of IR light to the sensor at different depths, there are potential advantages to addressing the difficulty of sEMG to detect deep muscles (Paleari et al., 2017). Furthermore, NIRS can potentially address the reduced recognition rate of EMG due to muscle fatigue and adjacent muscle signal scrambling (Nsugbe et al., 2020). Recent work has achieved an accuracy of 92.2% in an experiment to determine three-hand states using a combination of sEMG and NIRS, far exceeding the 73.3% accuracy of EMG alone in their experiments (Paleari et al., 2017). In addition, researchers have developed a new MMI to integrate the benefits of EMG signals and depth vision, which can automatically tag clusters of EMG data collection using depth vision without pre-tagging the data (Zhou et al., 2020). It has the potential for wide use in the operation of robotics and virtual reality applications. Moreover, sensor fusion means that the decoding steps become more complex since different sensors have different signal features, representing different physical meanings in the muscle activity. Optimizing algorithms to combine the signals more effectively from the various sensors is a point worth investigating. However, not all sensors can be combined and work together with each other. When designing fusion, it is essential to consider whether there is any interference between the combined sensors. For example, the EIT may interfere with the EMG or MMG, and the sound waves emitted by SMG may distort the PMG measurements. The sensor fusion also requires consideration of the sensor size and readout circuit integration. Since the readout circuits of different sensors are incompatible, integrating superabundant sensors makes the system very bulky and energy-intensive, which is fatal for wearable applications. The power consumption for recent works of each sensing technology is listed in Table 1. We must be careful that the power consumption for the presented works cannot be compared directly to the numbers. First, different works use different numbers of channels. And it is not easy to define a metric to compare the power consumption of each channel because multi-channel sensors can reduce power consumption by sharing ADCs. Second, power consumption is reported using different criteria: some works counted in wireless transmission and classifiers, while others include only the sensor’s own power consumption. So, power consumption is then compared in a more general way in Table 2. We could find that SMG normally consumes more power than other sensing technologies because they use active sensors that need to generate emitting energy. The same situation happens to PPG, EIT, and NIRS.

## Conclusion

This paper provides a comprehensive review of the state-of-the-art MMI sensors for potential super-resolution myography, categorized into three groups: biomechanical, biochemical, and bioelectrical, depending on the nature of signal sources. Super-resolution myography refers to the capability of both high-resolution and deep muscle detection, so we analyzed and discussed different MMI sensors in terms of their advantages and disadvantages with technical challenges separately. Some of these sensors have been explored and developed over many years. They have been studied in academia, while some recent sensor technologies are still in a proof-of-concept stage and require more investigation. We cross-sectionally compare these MMI sensors in terms of vulnerability and detectability that can be utilized for super-resolution myography. Four sources of interference, including tissue, interface, devices, and environment, are analyzed for all MMI sensors, which need careful consideration. High-density sensor array configuration is promising to improve myography’s resolution and real-time performance, whereas a more compact individual sensing unit allows more recording channels. In our comparison, MMG has the potential to achieve the greatest channel density, meeting super-resolution requirements (channel density larger than 10 cm^–2^; able to detect deep muscle activities). SMG can also achieve super-resolution; however, the active sensor unit and computation burden might bring higher power consumption and more latency, and thus it needs to be explored more. Both MMG and SMG are recent emerging sensors, and more studies are needed to make them become real super-resolution MMI. To interpret these recordings, a continuous decode model instead of pattern classification might offer a more natural and smoother control from MMI. However, the training process and cognitive burden may prevent it become more practical. Sensor fusion, which might facilitate researchers to complement the strengths of different sensing technologies, is also discussed for future development of super-resolution MMI. Finally, we conclude that emerging wearable myography with super-resolution will significantly facilitate MMI as control inputs for various application scenarios and yield unprecedented opportunities in neurotechnology, neurophysiology, neuroscience, and movement science.

## Author contributions

All authors listed have made a substantial, direct, and intellectual contribution to the work, and approved it for publication.

## Abbreviations

ADC: analog-digital converter
CNN: convolutional neural network
DOF: degrees of freedom
EEG: electroencephalography
EIT: electrical impedance tomography
EMG: electromyography
FEM: finite element method
FES: functional electrical stimulation
FMG: forcemyography
FSR: force sensing resistor
GMR: giant magnetoresistance
HD-FMG: high-density FMG
HIST: histogram
IR: infrared
iEMG: invasive EMG
KNN: k-nearest neighbors
LDD: longitudinal double differentiating
MeMG: mechanomyography
MMG: magnetomyography
MUAP: motor unit action potential
MUAPTs: motor unit action potential trains
MMI: muscle machine interface
NIRS: near-infrared spectroscopy
OMG: optomyography
OPM: optically pumped magnetometers
PCA: principal component analysis
PMG: phonomyography
PPG: photoplethysmography
RF: random forest
RMS: root-mean-square
RPTF: resistive polymer-thick-film
sEMG: surface EMG
SMG: sonomyography
SNR: signal-to-noise ratio
SQUID: superconducting quantum interference device
SVM: support vector machines
TMR: tunnel magnetoresistive.
